# Chiral and nematic phases of flexible active filaments

**DOI:** 10.1038/s41567-023-02218-w

**Published:** 2023-10-09

**Authors:** Zuzana Dunajova, Batirtze Prats Mateu, Philipp Radler, Keesiang Lim, Dörte Brandis, Philipp Velicky, Johann Georg Danzl, Richard W. Wong, Jens Elgeti, Edouard Hannezo, Martin Loose

**Affiliations:** 1 Institute of Science and Technology Austria (ISTA), Klosterneuburg, Austria; 2 WPI-Nano Life Science Institute, Kanazawa University, Kakuma-machi, Kanazawa, Japan; 3 Institute of Biological Chemistry, Faculty of Chemistry, University of Vienna, Vienna, Austria; 4 Theoretical Physics of Living Matter, Institute of Biological Information Processing and Institute for Advanced Simulation, Forschungszentrum Jülich, Jülich, Germany

**Keywords:** Biopolymers in vivo, Nanoscale biophysics, Computational biophysics, Supramolecular assembly

## Abstract

The emergence of large-scale order in self-organized systems relies on local interactions between individual components. During bacterial cell division, FtsZ—a prokaryotic homologue of the eukaryotic protein tubulin—polymerizes into treadmilling filaments that further organize into a cytoskeletal ring. In vitro, FtsZ filaments can form dynamic chiral assemblies. However, how the active and passive properties of individual filaments relate to these large-scale self-organized structures remains poorly understood. Here we connect single-filament properties with the mesoscopic scale by combining minimal active matter simulations and biochemical reconstitution experiments. We show that the density and flexibility of active chiral filaments define their global order. At intermediate densities, curved, flexible filaments organize into chiral rings and polar bands. An effectively nematic organization dominates for high densities and for straight, mutant filaments with increased rigidity. Our predicted phase diagram quantitatively captures these features, demonstrating how the flexibility, density and chirality of the active filaments affect their collective behaviour. Our findings shed light on the fundamental properties of active chiral matter and explain how treadmilling FtsZ filaments organize during bacterial cell division.

## Abstract (web summary)

Filaments of the FtsZ protein can form chiral assemblies. Now, active matter tools link the microscopic structure of active filaments to the large-scale collective phase of these assemblies.

## Main

In active systems, the emergence of large-scale order relies on a combination of local interactions between components and microscopic energy consumption. One typical property of such self-organizing systems is the spontaneous motility of their constituents. For example, individual proteins can polymerize into cytoskeletal filaments that can move due to asymmetric polymerization dynamics such as treadmilling or due to transport from the activity of motor proteins. Dynamic interactions between these active constituents can then lead to complex collective behaviour and phases not found at equilibrium, which have been under intense experimental and theoretical investigations in the past decade^1–7^. For example, reconstituted mixtures of actin or microtubule filaments with motor proteins self-organize into moving swarms, vortices and travelling waves^1,5,8^. The bacterial tubulin homologue FtsZ forms treadmilling filaments that can further organize into cytoskeletal patterns of moving bands and chirally rotating rings^9^. In living systems, such emergent behaviours can underlie a wealth of key biological phenomena such as cell division^10^, single and collective cell motility^11,12^ and organism morphogenesis^13,14^.

Active matter systems can be classified according to the symmetry of their constituents as either polar or nematic^15–17^. In addition, chiral active matter has recently attracted attention, where the constituents are either asymmetric in shape or perform a circular self-propelled motion. This includes curved cytoskeletal filaments, asymmetric synthetic swimmers or cell types displaying chiral motions on two-dimensional substrates^18–20^. How to relate the microscopic properties of active constituents to the large-scale outputs relevant for biology remains an outstanding challenge in the field^17^. For instance, the density of components as well as the degree of mutual attraction are often important control parameters in biological settings, which can affect the dynamic organization of active filaments and their function^10^. Furthermore, biological constituents are typically deformable, that is, they can change their shape as a function of external forces or crowding^6^. Altogether, despite increasing interest in chiral active matter at different scales^2,21–23^, how chiral constituents self-organize at different densities and interaction strengths, and how their local deformability contributes to large-scale collective features, remains poorly understood—both theoretically and experimentally.

FtsZ is an essential protein required for cell division in almost all bacteria, some archaeal and many photosynthetic eukaryotes^24–26^. It polymerizes into single-stranded filaments that grow from one end and shrink from the opposite end, driven by the hydrolysis of guanosine triphosphate (GTP) and a conformational change of the FtsZ monomer^27,28^. In vivo, these filaments first form a loose assembly at midcell, which quickly condenses into a tight ring-like structure called the Z-ring^29–32^. This cytoskeletal ring controls cell division by recruiting other cell division proteins to the midcell and by dynamically distributing them around the diameter of the cell. After the onset of constriction, the density of filaments in this ring further increases until the cell is split into two^29,33^. Although lateral interactions between treadmilling FtsZ filaments probably play an important role for the assembly and maintenance of the Z-ring in living cells as well as the emergence of cytoskeletal structures in vitro, the physical properties and interaction rules that give rise to their emergent patterns are currently not known. Here we characterized treadmilling FtsZ filaments at increasing surface densities and correlated their individual properties with their self-organization into large-scale patterns. In combination with active matter simulations, we elucidate the quantitative principles that govern the emergence of different collective cytoskeletal organizations from the local interactions of active constituents.

## Collective filament organization as a function of density

**Fig. 1:**
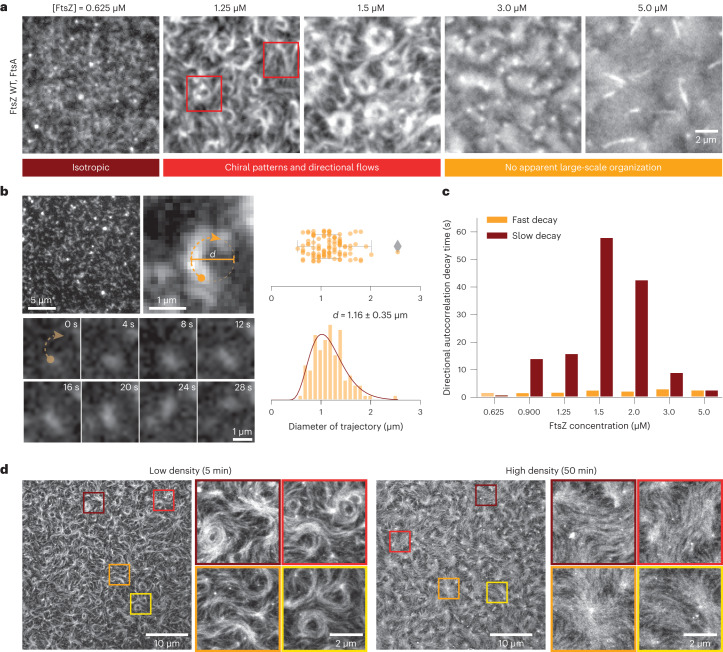
FtsZ filament organization changes with increasing density on SLBs. **a**, Representative TIRF micrographs of Alexa488-FtsZ at increasing FtsZ and constant FtsA concentrations. Below 0.625 µM FtsZ, filaments do not form higher-order structures. At 1.25 and 1.50 µM, FtsZ forms rotating rings and directionally moving filament bundles (see red squares). This organization is lost at concentrations above 3 µM FtsZ, at which filaments densely cover the membrane surface. The micrographs are representatives of at least four independent experiments (*n* = 4, 6, 8, 5 and 4 for [FtsZ] = 0.625, 1.25, 1.5, 3.0 and 5.0 µM, respectively). **b**, Representative images of a trajectory of a single FtsZ filament at 0.5 µM (left) and distributions of measured curvatures. The filament moves along a curved path, corresponding to an apparent diameter of 1.15 ± 0.35 µm (mean ± standard deviation, *n* = 105 trajectories from five independent experiments). **c**, Decay constants from fitting a biexponential function to directional autocorrelation curves from treadmilling trajectories. For intermediate concentrations, the best match is obtained by assuming a fast and slow decay constant (*n* = 4, 4, 6, 8, 4, 4 and 5 for [FtsZ] = 0.625, 0.900, 1.25, 1.50, 2.0, 3.0 and 5.0 µM, respectively; Extended Data Fig. 1d). **d**, Representative STED micrographs of 1.5 µM Atto633-FtsZ tethered to SLBs by 0.2 µM FtsA at low (5 min after starting the experiment (top)) and high (50 min (bottom)) densities. First, rotating rings and moving bundles coexisted on the membrane surface. With increasing filament density, the ring-like structures disappear. Source data

**Extended Data Fig. 1:**
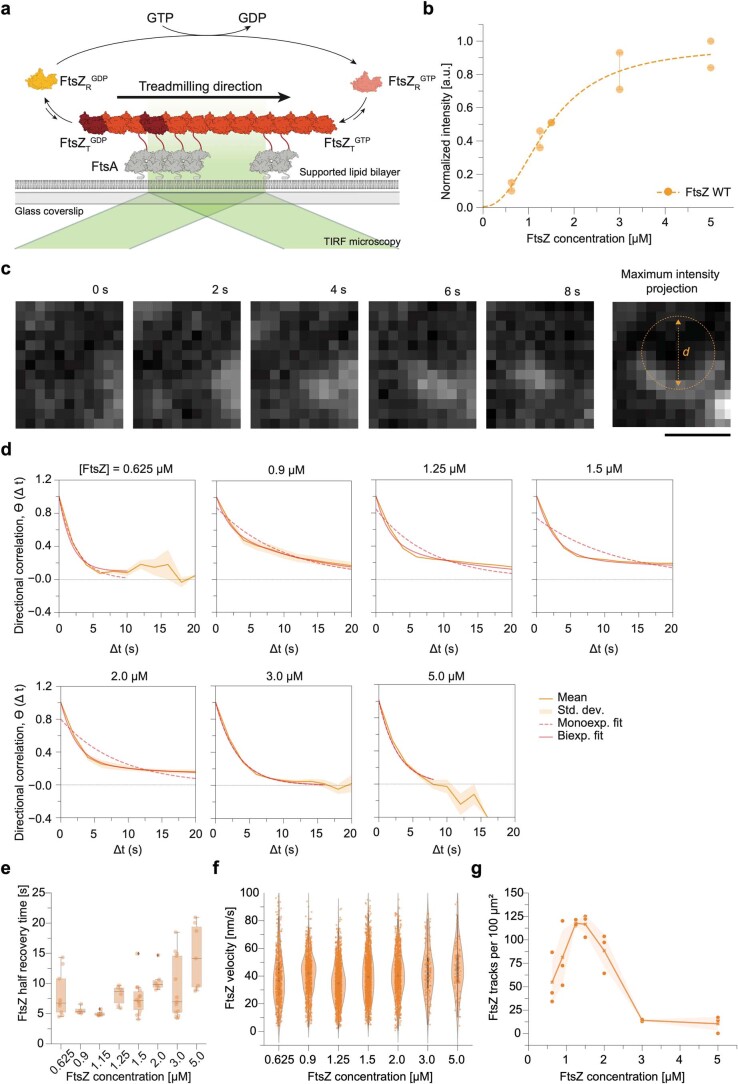
Characterization of FtsZ WT organization at increasing densities. **a**, Schematic illustrating experimental assay and FtsZ treadmilling. FtsZ filaments are recruited to the membrane surface by FtsA. TIRF microscopy allows visualizing filament polymerization dynamics. **b**, Quantification of the intensity of Alexa488-FtsZ WT during a TIRF titration experiment. The density of FtsZ filaments on the supported membrane is saturated at 3 µM (n = 2 for each FtsZ concentration). **c**, Snapshots of time lapse movie of a single FtsZ filament treadmilling on a membrane surface. The maximum intensity projection reveals the curved trajectory of the filament. **d**, Representative fits of mono- and bi-exponential functions to the directional autocorrelation of treadmilling trajectories. Also see Fig. 1c. **e**, Quantification of the membrane residence time of FtsZ WT by FRAP experiments with increasing bulk concentrations (n = 9, 4, 6, 6, 14, 6, 13 and 7 for [FtsZ] = 0.625, 0.9, 1.15, 1.25, 1.5, 2, 3 and 5 µM). The boxes in **e** indicate the 25–75th percentiles, whiskers show the maximum/minimum values within the standard deviation and the midline indicates the median. **f**, Quantification of the FtsZ velocity at increasing concentrations by differential imaging and speckle tracking. (N = 3 independent experiments for each concentration, n = 658, 977, 1412, 1400, 1061, 170 and 126 trajectories were analysed for [FtsZ] = 0.625, 0.9, 1.25, 1.5, 2, 3 and 5 µM). The dot in f indicates the mean and the thick black line the 25–75th percentiles. **g**, Number of FtsZ treadmilling trajectories within a 100 µm^2^ area after differential imaging. Dots represent the mean track number of independent experiments; the line indicates the mean of all experiments and the shaded area represents the SEM. Source data

To understand the properties of FtsZ filament–filament interactions, we first experimentally explored the phase space of the possible organizations of FtsZ filaments as a function of their density. We used a previously established in vitro reconstitution assay^9^, where treadmilling FtsZ filaments are recruited to the surface of a supported bilayer by its physiological membrane anchor FtsA (Extended Data Fig. 1a). Using total internal reflection fluorescence (TIRF) microscopy, we recorded the emergent behaviour of the membrane-bound filaments. We found that we could control the density of membrane-bound filaments by changing the protein concentration in the buffer solution. When we gradually increased the FtsZ bulk concentration from around 0.6 to 5.0 µM and maintained the FtsA concentration constant, the fluorescence intensity of FtsZ on the membrane increased linearly until it saturated at concentrations higher than 3.0 µM (Extended Data Fig. 1b). With increasing density, we found the large-scale organization of filaments to change (Fig. 1a and Supplementary Video 1): at FtsZ concentrations lower than 0.6 µM, individual filaments travelled across the membrane surface via treadmilling^9^. Fluorescence microscopy could not directly reveal their intrinsic curvature of individual filaments, but the maximum intensity projections of the time-lapse videos showed that their trajectories followed a curved path corresponding to a circle with a diameter of 1.16 ± 0.35 µm and heavily biased in the clockwise direction (Fig. 1b and Extended Data Fig. 1c). Above 0.60 µM, at FtsZ bulk concentrations of 1.25 and 1.50 µM, FtsZ filaments organized into chiral rotating rings that persisted for around 5–6 min and coexisted with comet-like structures and moving bundles of treadmilling filaments^9^. To quantify the directionality of the observed cytoskeletal flows, we calculated the corresponding directional autocorrelation^34,35^. Consistent with the persistent, long-range polar motion of treadmilling filament bundles, we found a long decay time of 16 and 68 s at 1.25 and 1.50 µM FtsZ, respectively (Fig. 1c and Extended Data Fig. 1d). In fluorescence recovery after photobleaching (FRAP) experiments, we found that the mean lifetime of FtsZ monomers in filaments was only around 7 s (Extended Data Fig. 1e), confirming that their directional motion is a collective property of the system. At higher bulk concentrations, the cytoskeletal organization on the membrane changed, as seen in a previous study using an artificial construct of autonomously membrane binding FtsZ (ref. ^36^). Filaments now densely covered the membrane surface without apparent large-scale organization or directional flows (Fig. 1a). Indeed, our directional autocorrelation analysis showed that the long decay time at high filament densities was absent (Fig. 1c). Despite the absence of long-range flows, however, FtsZ filaments were still dynamic: first, we found that at these high densities, FtsZ still treadmills at a similar velocity, even though the number of identifiable tracks dropped substantially (Extended Data Fig. 1f,g). Second, FtsZ filaments still continuously exchanged monomers, although the recovery half-time was increased to around 15 s (Extended Data Fig. 1e).

**Extended Data Fig. 2:**
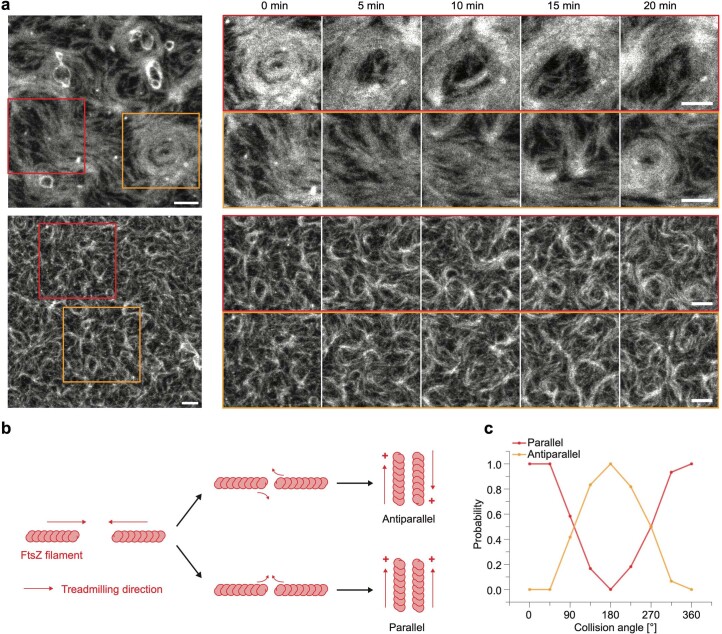
STED microscopy. **a**, Representative micrographs of STED time-lapse experiments of Atto633-FtsZ WT. The insets show either rings which dynamically re-arrange into bundles (red) or bundles, which re-arrange into rings (orange). Scale bars are 1 µm. **b**, Model drawings showing the different types of collisions FtsZ filaments can undergo. In the aftermath of collision events FtsZ filaments align either parallel or antiparallel. **c**, Probability of parallel and antiparallel alignment as a function of collision angle. 151 collision events in 6 independent experiments were quantified. Source data

Despite the obvious differences in their large-scale organization at different filament densities, conventional TIRF microscopy was not able to reveal the underlying mechanism of this phase transition. We, therefore, applied fast time-lapse imaging using stimulated emission depletion (STED) microscopy. The increased resolution helped to visualize that rotating rings and moving bands comprise many transiently interacting FtsZ filaments (Fig. 1d, Supplementary Video 2 and Extended Data Fig. 2a). The apparent weak interactions between filaments suggest that their local polar orientation is an emergent property of the ensemble, rather than the result of specific static interactions, such as residue contacts between individual filaments (Extended Data Fig. 2b,c).

Together, these observations and quantifications demonstrate a density-dependent transition in the large-scale organization of treadmilling FtsZ filaments. Although STED microscopy observations strongly suggest that filament alignment is the result of weak, transient interactions between them, it could not yet reveal the mechanism underlying this transition.

## Collective filament organization as a function of bending rigidity and attraction

Given our experimental findings that single FtsZ filaments displace along curved, chiral paths (Fig. 1b), and previous theoretical work showing that chiral self-propelled filaments could organize into ring-like phases at intermediate densities in the absence of attraction^2^, we explored whether a minimal coarse-grained model could quantitatively reproduce the observed phenomenology. We modelled this system on a mesoscopic level by a collection of overdamped, self-propelled semiflexible filaments on a two-dimensional surface^37–39^, systematically changing the strength of attractive interactions, filament densities and bending rigidities (Methods provides more details on the simulation framework).

**Fig. 2:**
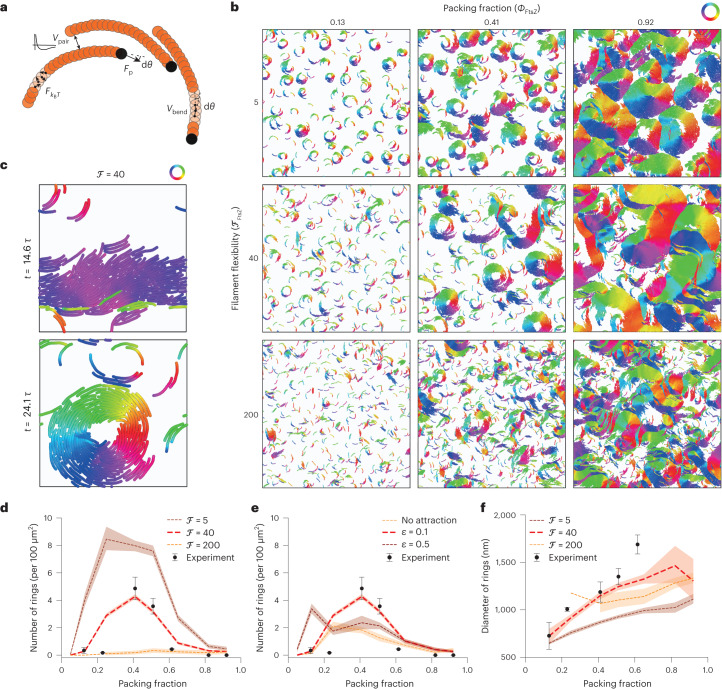
Collective FtsZ WT self-organization as a function of bending rigidity and attraction. **a**, Scheme of the simulation model. **b**, Phase diagram of the large-scale patterns (*L* = 212*d*, corresponding to the field of view in Fig. 1a) with varying filament flexibilities (measured by flexure number ℱ; vertical axis) and densities (horizontal axis). Filaments are colour coded according to the orientation of the bond vectors between beads. We observe the ring-like self-organization of rigid filaments (ℱ = 5), spatial coexistence of chiral rings and polar bands in the regime of semiflexible filaments (ℱ = 40) and disordered patterns with flexible filaments (ℱ = 200). **c**, Temporal coexistence of chiral rings and polar bands in a small simulated system (*L* = 42*d*) of intermediate density (*Φ* = 0.5) and filament flexibility (ℱ = 40). Filaments are colour coded according to the orientation of bond vectors between beads. **d**–**f**, Quantitative comparison of ring density and diameter between simulations and experiments (Methods provides details on the quantification and comparisons). The red solid line corresponds to the best match of filament flexibility and attraction (ℱ = 40, *ε* = 0.1*k*_B_*T*). The number of rings was determined from 4, 4, 9, 11 and 3 independent experiments at *Φ* = 0.12, 0.23, 0.40, 0.50 and 0.60. The ring diameter was measured from *n* = 27, 15, 63, 65 and 17 randomly chosen rings out of the experiments at increasing packing fractions. The dotted lines represent the mean; the shaded area, the 95% confidence interval; and the error bars of experimentally determined values, the standard deviation. Source data

Each semiflexible, coarse-grained filament is simulated as a worm-like chain, with bending rigidity described by the potential *V*_bend_, and is self-propelled with tangential force **F**^*i*^_p_ to describe an effective treadmilling velocity ${v}_{0}={F}_{{\rm{p}}}^{i}/\gamma$, where *γ* is the friction coefficient with the membrane. In the following, we use the flexure number^28^ ℱ, defined as the ratio of self-propulsion forces to bending rigidity ${\mathcal{F}}{{=}}\frac{{f}_{{\rm{p}}}{L}_{{\rm{f}}}^{3}}{{k}_{{{\rm{bend}}}}}$, as a measure of filament flexibility (inverse of rigidity where *f*_p_ is the self-propelled force by unit length, see Methods). To account for the observed chirality, filaments are considered to have spontaneous signed curvature with rest angle d*θ*. An effective thermal noise force ${F}_{{k}_{{\rm{B}}}T}^{i}$ accounts for the many sources of noise in the system (Fig. 2a). Finally, based on previous studies of FtsZ filament–filament interactions^32,40,41^ and our own observations (Fig. 1d), we first considered mid-range attractive interactions between filaments (*V*_pair_).

**Extended Data Fig. 3:**
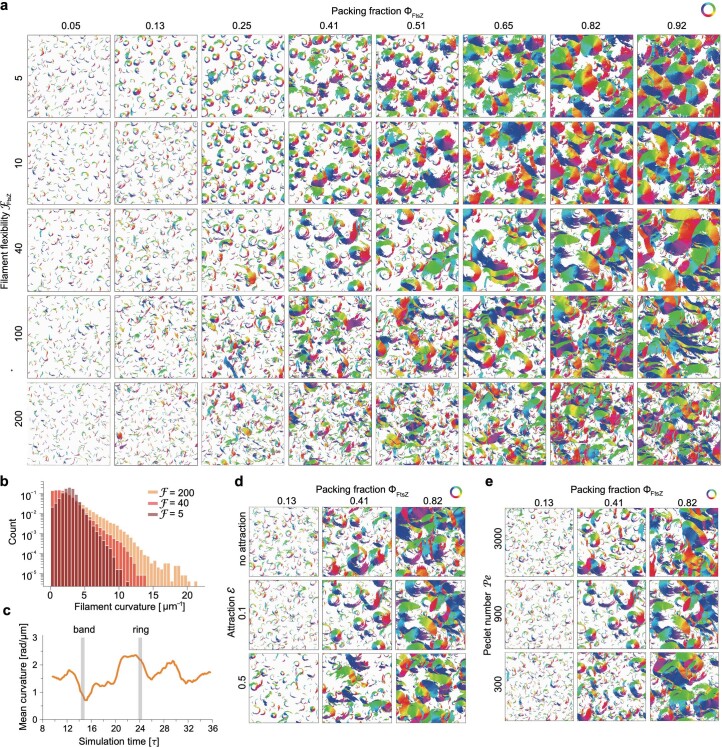
Numerical model of FtsZ WT - self-organization with varying flexibility, attraction and noise. **a**, Extended visual phase diagram of the large-scale patterns (*L* = 212 *d*) with varying filament flexibility (measured by flexure number ℱ, vertical axis) and density (horizontal axis). **b**, Distribution of filament curvatures for rigid (ℱ = 5), semiflexible (ℱ = 40) and flexible filaments (ℱ = 200). Only rigid filaments keep a well-peaked curvature distribution. **c**, Evolution of average curvature of the system showing the temporal coexistence of chiral rings and polar bands in a small system size (*L* = 42 *d*) of intermediate density (*Φ* = 0.5). The vertical lines represent the timepoints of the snapshots in Fig. 2c. **d**, Visual phase diagram of the large-scale patterns (*L* = 212 *d*) with varying attraction (vertical axis) and density (horizontal axis). The rings are less stable without lateral attraction, while with the attraction being too strong, rings are favoured in small density (*Φ* < 0.25), while in the intermediate density cluster formation is very rapid resulting in lower occurrence of rings. **e**, Visual phase diagram of the large-scale patterns (*L* = 212 *d*) with varying noise (measured by Peclet number *Pe*, vertical axis) and density (horizontal axis). The rings are more abundant and stable with higher Peclet numbers, with strongest differences at lower density (0.05 < *Φ* < 0.25). Filaments are colored according to the orientation of the bond vectors between beads. Source data

We observed that the large-scale organization of FtsZ filaments at different densities was strongly affected by the filament flexibility (Fig. 2b, Extended Data Fig. 3a and Supplementary Video 3). For very rigid, curved filaments (ℱ = 5), ring-like patterns and vortices dominated the system throughout the explored density range, thus confirming a previous study^2^. At the other extreme, filaments with very low bending rigidity (ℱ = 200) easily deformed and rarely formed chiral rings, which were unstable at all the tested densities. However, for curved, semiflexible filaments (ℱ = 40), we found two density-driven transitions as in our experiments. Moreover, in the intermediate-density range, we could observe a coexistence—both temporal and spatial—of chiral rings and straighter travelling bands characterized by a low spontaneous curvature. More quantitatively, decreasing the bending rigidity resulted in the loss of a well-peaked filament curvature distribution (Extended Data Fig. 3b). Interestingly, this was in strong qualitative agreement with our experimental observations, where filaments can adopt a wide range of curvatures even at intermediate densities^34^ and where rings are interspersed with less ordered filament assemblies (Fig. 1a,d and Extended Data Fig. 2a). In addition, as in our experimental data, we observed dynamical interconversion between ring and band patterns (Fig. 2c, Extended Data Fig. 3c and Supplementary Video 4).

We also investigated the effect of filament attraction (*ɛ*) and thermal noise (Peclet number) on the phase diagram. For low attraction, we systematically observed a transition from disordered patterns to rings above a critical density, as previously reported^2^. Interestingly however, for strong filament attraction, this transition was largely lost, with stable rings able to form even at the lowest experimental densities (Extended Data Fig. 3d), similar to the case of very rigid filaments. From a physical perspective, this is due to ring formation being energetically favoured, instead of being an active kinetic state in the case of purely repulsive self-propelled filaments^3,5^. Large changes in the Peclet number also affected the first transition, as well as the overall density of rings in the system (Extended Data Fig. 3e). Above a second density threshold, we could observe a loss of ring patterns in all of the cases.

Together, this phase diagram and the observed differences in filament organization argue that filament flexibility and density could be the key parameters for FtsZ self-organization.

## Quantitative comparison between model and experiments

To more systematically and quantitatively compare the simulations and experiments, we first sought to constrain the model parameters. From single-filament trajectories (Fig. 1b), the result of our treadmilling analysis (Extended Data Fig. 1f) and previously published values^9,35^, we considered a treadmilling speed of *v*_0_ = 0.04 μm s^–1^, and estimated the packing fraction of filaments *ϕ* based on our calibration experiments (Extended Data Fig. 1b). The filament aspect ratio in our mesoscopic simulations corresponded to a length of 400 nm and effective thickness of 50 nm to account for the loose organization of filaments on the membrane^42^ (Fig. 1d). The estimation of filament chirality based on the curvature of single-filament trajectories was limited by the resolution of fluorescence microscopy, but still allowed us to guide the parameter space by estimating d*θ* to yield the single-filament rotation diameter of 600–1,000 nm. Furthermore, from the variability of this rotation diameter, we could estimate the Peclet number to be in the range of ~700–1,300, a range between which the effect of Peclet number on global dynamics is limited (Extended Data Table 1 lists a summary of the parameter values and associated experimental constraints). Therefore, after this estimation, we were left with only two free parameters, which we systematically explored in our simulations (in addition to density): the adhesion strength *ε* and the filament flexibility measured by flexure number ℱ.

**Extended Data Fig. 4:**
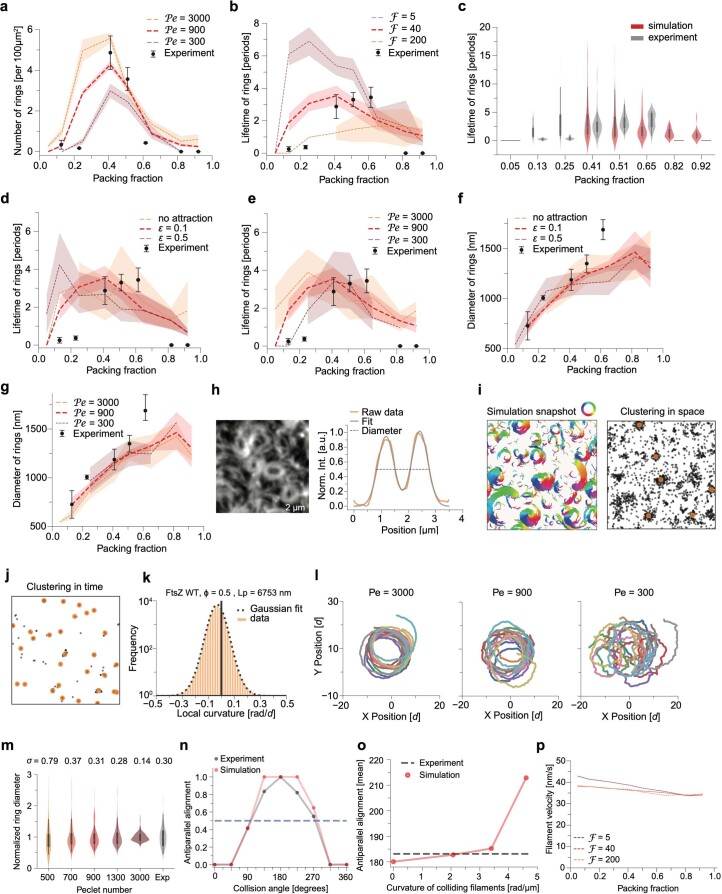
Numerical model of FtsZ WT - quantitative analysis and parameter refinement. **a-b, d-g**, Quantitative comparison of ring density (**a)**, lifetime (**b,d,e**) and diameter (**f,g**) between large-scale simulations with varying parameters (flexure number, filament attraction and Peclet number) and experiments. The thick red line corresponds to the best match of simulation parameters to experiments. Dotted lines represent the mean, the shaded area the 95% confidence interval and the error bars of experimentally determined values represent the standard deviation. The number of rings was determined from 4, 4, 9, 11 and 3 independent experiments at *Φ* = 0.12, 0.23, 0.4, 0.5 and 0.6. The lifetime was quantified from 18, 24, 16, 33 and 24 rings, while the ring diameter was measured from n = 27, 15, 63, 65 and 17 randomly chosen rings from experiments at increasing packing fractions. **c**, Comparison of ring lifetime distributions between the simulations (ℱ = 40) and experiments at corresponding densities. The lifetime from experimental rings was quantified from 18, 24, 16, 33 and 24 rings. The thick black line extends from the mean and covers the range from the 25–75th percentile. **h**, Representative micrograph of TIRF experiment and the quantification of ring diameter. The line profile on the right corresponds to the orange dashed line. **i**, Example illustrating the automatic detection of rings. Left box depicts a simulation snapshot with filaments forming rings and bands (with filaments are colored according to the orientation of the bond vectors between beads), while the right box shows the result of clustering of filament rotation centers in space after applying all cutoffs (see Material & Methods). **j**, Result of the ring detection algorithm for the whole simulation after applying the time clustering algorithm to detect the rings in all calculated simulation snapshots. **k**, Analysis of filament persistence in the numerical simulations based on the distribution of local curvatures. Persistence length was extracted from variance of Gaussian function fitted to the data. **l**, Single filament trajectories (10 independent simulations) with varying Peclet number. **m**, Comparison of distributions of single-filament trajectories analysed in Fig. 1b and simulations with different Peclet numbers. 100 rings of each Peclet number were analysed in simulations, 105 rings were analysed in experiments. The thick black line extends from the mean and covers the range from the 25–75th percentile. **n**, Probability of antiparallel alignment of FtsZ bundles as a function of collision angle in experiment (corresponds to Extended Data Fig. 2c) and simulations. **o**, Mean of antiparallel alignment distribution as a function of filament curvature in simulations. **p**, Treadmilling velocities of single filaments from simulations at different densities and flexure number. Source data

Interestingly, intermediate values of filament flexibility, as well as low to intermediate attraction, provided a good match for a number of qualitative and quantitative features of our dataset. First, we could quantitatively reproduce not only the two thresholds of appearance and disappearance of rings as a function of density but also the absolute probability of ring formation in the intermediate-density region (Fig. 2d,e and Extended Data Fig. 4a). Strikingly, when measuring the ring lifetime (normalized by a period of filament rotation) in both experiments and simulations, we found excellent agreement with our predicted parameter regime (Extended Data Fig. 4b–e). Finally, our simulations predicted that the ring diameter should monotonously increase with density, a feature we found to agree with our data (Fig. 2f and Extended Data Fig. 4f,g). Overall, this demonstrates that the transitions seen in the data as a function of density can be quantitatively explained by a simple theoretical framework of flexible chiral active filaments. Interestingly, this analysis suggests FtsZ filaments being much more flexible than previously anticipated^2,38^. This prompted us to theoretically understand how conformational changes in the FtsZ filament occur at the microscopic level as a function of increasing density, as well as experimentally test it more directly.

## Mechanism of the chiral to nematic transition

**Fig. 3:**
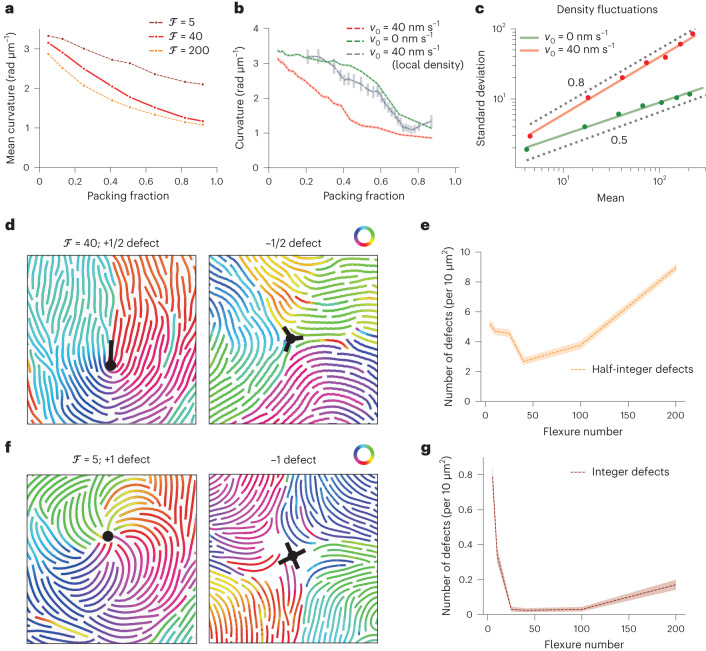
Mechanisms of active filament straightening and chiral to nematic transition. **a**, Average filament curvature with varying densities, showing density-driven straightening (large-scale simulations correspond to snapshots and analysis in Fig. 2b,d,f; *L* = 212*d*). The solid red line corresponds to the best match of flexure number from the quantitative comparison shown in Fig. 2. **b**, Average filament curvature as a function of packing fraction in active and passive systems (*L* = 42*d*). Although active filaments gradually straighten with increasing density (red), passive filaments (*v*_0_ = 0 nm s^–1^) straighten more abruptly above a packing fraction of 0.5–0.6 (green). Plotting the active filament curvature as a function of local density (Methods) displays a similar trend of straightening as in the passive system (binned scatter plot, grey). The filament curvature versus local density was analysed over the whole simulated density range (*Φ* = 0.05–0.90). The dotted lines represent the mean and the shaded area, the 95% confidence interval. **c**, Scaling of density fluctuations for passive (*v*_0_ = 0 nm s^–1^, green) and active (red) filaments (*L* = 42*d*, *Φ* = 0.4), showing the predicted scaling from equilibrium (exponent, 0.5) and giant density fluctuations (exponent, 0.8), respectively. **d**,**f**, Nematic and integer defects in high density (*Φ* = 0.95, *L* = 42*d*) of semiflexible (ℱ = 40) (**d**) and rigid (ℱ = 5) (**f**) filaments. Only bonds of the filaments (without the full diameter of beads) are presented for clarity. Filaments are colour coded according to the orientation of the bond vectors between beads. **e**, Density of nematic defects as a function of filament flexibility (*Φ* = 0.9, *L* = 42*d*). Defect density first decreases with increasing filament flexibility and then increases beyond a flexure number of 50. **g**, Density of integer defects as a function of filament flexibility (*Φ* = 0.9, *L* = 42*d*). Defect density sharply decreases with increasing filament flexibility. The dotted lines represent the mean and the shaded area, the 95% confidence interval. Source data

**Extended Data Fig. 5:**
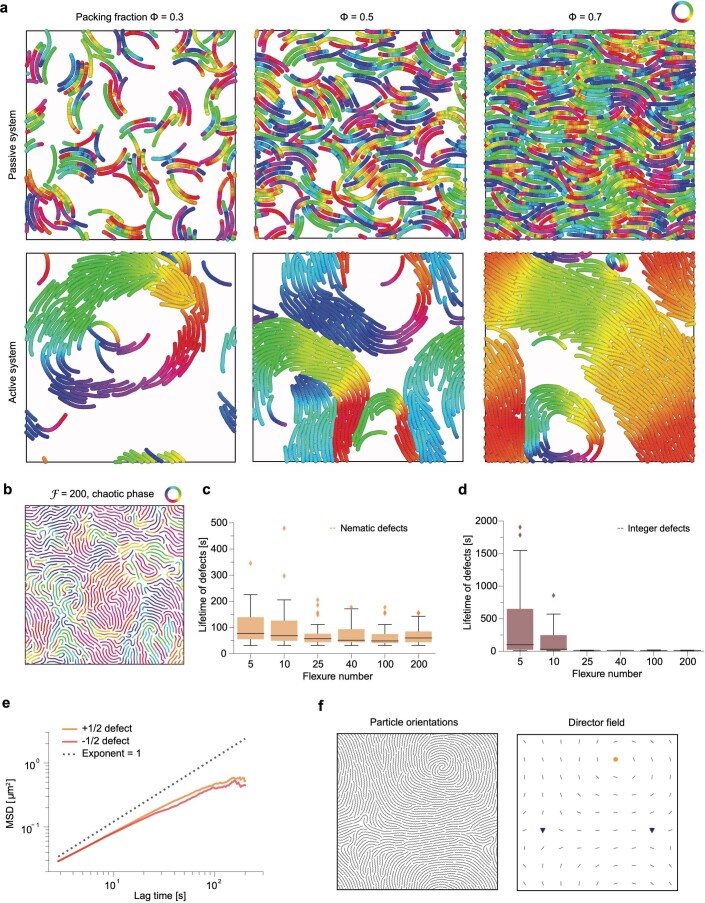
Analysis of active vs. passive filament straightening and topological defects. **a**, Snapshots of simulations of passive (v_0_ = 0 nm s^–1^) and self-propelled filaments in 3 different densities (Φ = 0.3, 0.5, 0.7; L = 42 *d*). Active filaments show visibly stronger density fluctuations than passive filaments. **b**, Phase of highly flexible filaments with increased density of topological defects. Filaments are colored according to the orientation of the bond vectors between beads. Only bonds of the filaments (without the full diameter of beads) are presented for clarity. **c**, Lifetimes of nematic defects with varying flexure number (n = 41, 47, 61, 33, 44 and 64 for ℱ = 5, 10, 25, 40, 100 and 200). **d**, Lifetime of integer defects with varying flexure number (n = 22, 15, 3, 1, 4 and 4 for ℱ = 5, 10, 25, 40, 100 and 200). The boxes indicate the 25–75th percentiles, whiskers show the maximum/minimum values within the standard deviation and the midline indicates the median. **e**, Mean squared displacements (MSD) of nematic defects detected in the simulations (all flexure numbers combined), showing sub-diffusive scaling. The MSD of +1/2 defects is slightly higher, but the overall MSD in real time and space units is relatively small (0.4 µm^2^ at 100 s). **f**, Snapshot of high-density simulation (Φ = 0.95, ℱ = 10) showing the nematic director field (right) analysed from orientations of each polymer bead (left). Yellow circle represents identified +1 spiral defect, whereas blue triangles correspond to +1/2 defects. Source data

We first reasoned that the increase in ring diameter and ultimate loss of rings with increasing density originates from the straightening of the semiflexible filaments. Indeed, we found that the average filament curvature smoothly decreased with increasing density in our simulations, a decay that was most pronounced for more flexible filaments (Fig. 3a). Since such straightening could arise from steric interactions as a purely passive effect, we performed similar simulations at different densities but for non-treadmilling filaments (*v*_0_ = 0 μm s^–1^; Extended Data Fig. 5a). Interestingly, although we still found filament straightening, filament curvature changed only slightly up to a packing fraction of 0.5–0.6, before abruptly dropping, unlike in the active case where straightening is gradual (Fig. 3b). Seeking to understand the discrepancy between active and passive straightening, we noticed that activity creates giant density fluctuations (a classical result for self-propelled rods^43^), and reasoned that this would result in most filaments experiencing a much larger local density than the average (Fig. 3c). Importantly, when plotting local filament curvature versus local density in active systems, we could recover a trend much closer to the equilibrium system (Fig. 3b shows the curvature versus global density). This indicates that although straightening can occur via a passive steric effect, its strength and density dependence is strongly modified by activity via the effect of self-propulsion on density fluctuations.

Next, we sought to better characterize the transition by quantitatively studying how the local orientational order and associated topological defects were affected by filament flexibility and density. Interestingly, we found the spontaneous appearance of +1/2 and −1/2 topological defects at high densities in the whole range of filament flexure numbers, although their statistics were strongly affected by the level of filament flexibility (Fig. 3d,e, Supplementary Fig. 5c and Supplementary Video 5). Although the density of nematic defects first decreased with increasing filament flexibility due to filament straightening, very flexible filaments (with their wide distribution of local curvatures allowing many sharp turns) entered a phase characterized by a strong increase in the density of nematic defects with a smaller lifetime (Extended Data Fig. 5b). Finally, in the regime of low filament flexibility, we found that individual filaments were still highly curved and self-organized into spiral patterns, even above the density where well-defined rings disappeared. These were characterized by the presence of stable +1 and −1 topological defects and displayed chiral rotation dynamics (Fig. 3f,g, Extended Data Fig. 5d and Supplementary Video 5). To sum up, the analysis of microscopic filament properties revealed that filaments enter different regimes in high density with varying flexure numbers: although rigid filaments preserve their chirality, semiflexible filament straightening results in a nematic-like phase due to the combined effect of activity and steric interactions.

## HS-AFM imaging of dynamic FtsZ filaments

**Fig. 4:**
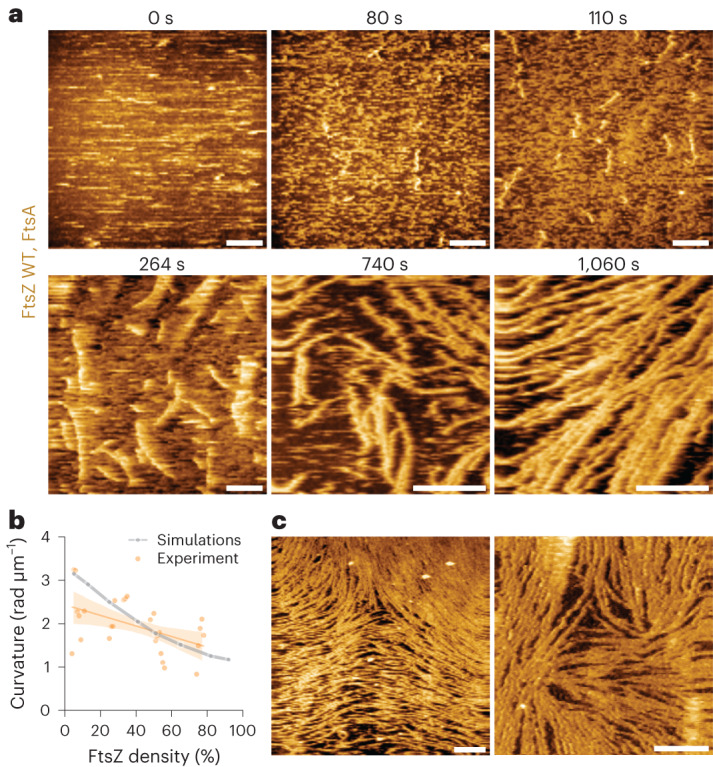
HS-AFM imaging shows nematic organization at high densities of FtsZ filaments. **a**, Representative HS-AFM time-lapse experiment, showing an increase in the density of FtsZ WT filaments with time. Scale bars, 500 nm. **b**, Curvature of FtsZ WT filaments at increasing densities from experiments (orange; mean ± standard deviation) and simulations (grey). With increasing densities, the curvature of individual filaments decreases. The curvature of FtsZ filaments was quantified from five independent experiments. The dots indicate the mean curvature of all the filaments at the indicated packing fraction. The thick lines represent the mean and the shaded area, the 95% confidence interval. **c**, At high densities, FtsZ WT filaments show nematic order with topological defects ([FtsZ] = 1.5 µM (left), 4.0 µM (right)). Scale bars, 500 nm. Source data

To test our model’s prediction of filament straightening as a function of density, we needed to image individual filaments at different filament densities. As fluorescence microscopy could not visualize the properties of individual treadmilling FtsZ filaments within large-scale cytoskeletal patterns (Fig. 1), we used high-speed atomic force microscopy (HS-AFM)^44,45^. At low densities, HS-AFM showed individual filaments that travelled across the membrane surface and rapidly moved in and out of the field of view, which made it difficult to track individual filaments over time. During the course of the experiments, filaments increased in density, but neighbouring filaments did not form stable lateral contacts; instead, they displayed fast lateral fluctuations on the membrane surface (Fig. 4a and Supplementary Video 6). This suggests that the attraction energy was comparable or smaller than thermal fluctuations, consistent with our numerical simulations. HS-AFM also allowed us to quantify the filament shape as a function of density, which was impossible using data from TIRF and STED microscopy. Interestingly, we found a gradual decrease in filament curvature as a function of overall surface density, which effectively matched our theoretical prediction for semiflexible filaments (Fig. 4b). At the highest densities, where we observed homogeneous fluorescence intensity with TIRF microscopy (Fig. 1a), HS-AFM revealed hallmarks of active nematics as in our simulations, that is, half-integer topological defects that spontaneously formed (Figs. 3d and 4c). We observed a mean density of 1.0 ± 0.4 defects per 10 µm^2^ (*n* = 4), a value closest to the simulations of semiflexible filaments with ℱ = 40 (Fig. 3e). We never observed the integer defects predicted for very rigid filaments (Fig. 3f) nor the extreme curvature values predicted for very flexible filaments, providing additional evidence for FtsZ semiflexibility (Methods). Defects showed limited mobility and typically persisted longer than our observation time, as in simulations run for comparable timescales (Extended Data Fig. 5e). However, they were able to merge and annihilate each other (Supplementary Video 6). Individual filaments had no discernable intrinsic curvature. In conclusion, these experimental observations confirm our theoretical prediction that filament organization at high densities is governed by nematic, rather than chiral, symmetry due to the straightening of FtsZ filaments.

## Predicting filament organization in mutant conditions

**Fig. 5:**
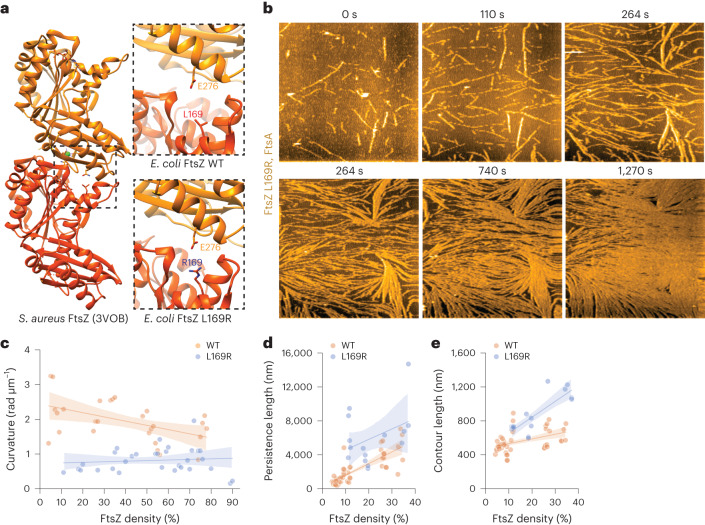
Point mutation in FtsZ L169R changes the properties of FtsZ filaments. **a**, Ribbon model of the *S. aureus* FtsZ filament (PDB 3VOB) (left) and the longitudinal interface predicted for *Escherichia coli* FtsZ WT (right, top) and L169R (right, bottom). The leucine to arginine mutation probably enables a novel salt bridge, which stabilizes the FtsZ filament. **b**, Representative HS-AFM time-lapse experiment, showing an increase in density of FtsZ L169R filaments. Already at low densities, the mutant filaments are less dynamic and more rigid. Scale bars, 500 nm. **c**, Curvature of FtsZ L169R filaments as a function of density is lower than FtsZ WT filaments at all the tested densities. **d**, Persistence length of FtsZ L169R filaments as a function of density, showing a two times higher value compared with FtsZ WT. **e**, Contour length of FtsZ L169R as a function of density, increasing faster than FtsZ L169R. Data shown in **d** and **e** are taken from experiments at low densities (<40%), as FtsZ L169R filaments at higher densities can be longer than the field of view and impede quantification. The curvature, persistence and contour length of FtsZ filaments were quantified from 5 (WT) and 4 (L169R) independent experiments. The dots indicate the mean result of all the filaments at the indicated packing fraction. The thick lines represent the mean and the shaded area, the 95% confidence interval. Source data

**Extended Data Fig. 6:**
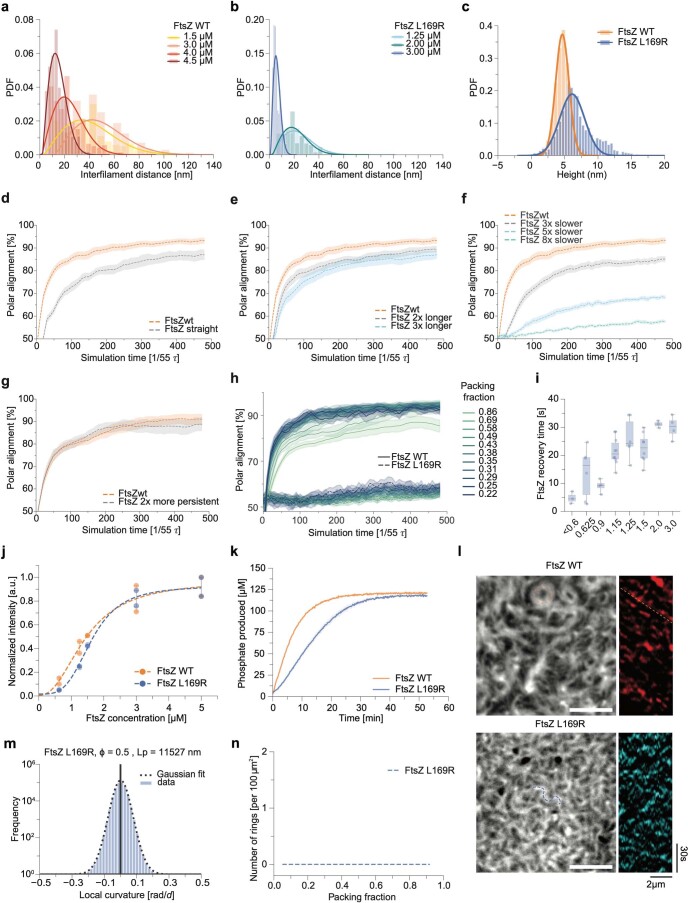
Characterization of FtsZ L169R and effect of filament properties on polarity sorting. **a, b**, Interfilament distance of FtsZ WT (a) and FtsZ L169R (b) (n[WT] = 2, 2, 2 and 3 for 1.5, 3, 4 and 4.5 µM and n[L169R] = 5, 3 and 1 for 1.25, 2 and 3 µM). **c**, Height distribution of FtsZ WT and L169R filaments (n = 5 and 3 for FtsZ WT and L169R respectively).**d-g**, Effect of individual filament properties on the polarity sorting. In each plot, simulations with FtsZ WT are presented to be compared with simulations of FtsZ WT with one changed parameter. All studied properties decrease the polar sorting with persistence length having the smallest effect. Each plot represents averaged results over four packing fractions in range to *ϕ* = 0.29 to *ϕ* = 0.88. Lines represent the mean and the shaded area the 95% confidence interval. **h**, Polarity alignment of simulations with FtsZ WT and L169R in individual densities. The analysed simulations correspond to Fig. 6b. **i**, Quantification of FtsZ L169R membrane residence time by FRAP experiments. Dots represent individual experiments (n = 3, 6, 4 10, 6, 8, 3 and 4 for [FtsZ] = <0.6, 0.625, 0.9, 1.15, 1.25, 1.5, 2 and 3 µM). Boxes indicate the 25–75th percentiles, whiskers indicate the maximum/minimum values within the standard deviation and the midline shows the median. **j**, Quantification of Alexa488-FtsZ L169R TIRF intensities at increasing concentrations (n = 2 for each FtsZ concentration). **k**, Quantification of the GTPase hydrolysis rate of 5 µM FtsZ WT or L169R. The rates are 2.2 ± 0.03 and 0.78 ± 0.03 GTP/FtsZ/min respectively (mean ± s.d. p-value: 9.27*10-4, 2-tailed, equal variance t-test; n = 2). Lines represents the mean and the shaded area the standard deviation. **l**, TIRF micrographs and representative kymographs after differential imaging for FtsZ WT and L169R. Kymographs were obtained along the dashed lines. No directional polymerization is observed for L169R. Scale bars are 2 µm. **m**, Distribution of local curvatures in simulations of FtsZ L169R. Persistence length was extracted from variance of Gaussian function fitted to the data. **n**, Analysis of number of rings in simulations with FtsZ L169R using the same ring detection algorithm as for FtsZ WT (Extended Data Fig. 4i, j). Source data

Our theory suggests that single-filament properties (such as semiflexibility and chirality) can explain the complex density-dependent transitions observed experimentally, providing a link from microscopic properties to ring self-organization observed in vivo. Given that a number of FtsZ mutants had previously been described to crucially affect ring formation in vivo, we sought to test whether we could explore, in vitro and in silico the relationship to single-filament properties. Looking for filaments either with lower curvature or enhanced bending rigidity, we reviewed previously described FtsZ mutants and identified FtsZ L169R as an interesting candidate. This mutant protein was previously described to have increased lateral interactions, as the protein shows enhanced pelleting in in vitro assays and more stable Z-rings in vivo^42,46^. We performed AlphaFold^47,48^ predictions of an FtsZ L169R dimer using the crystal structure of FtsZ filaments from *Staphylococcus aureus* as a template^49^. The positively charged arginine residue at position 169 is in fact located at the longitudinal interface, facing towards a negatively charged glutamic acid residue at position 276 of the neighbouring monomer (Fig. 5a). We reasoned that the L169R mutation could result in an additional salt bridge between two monomers in the filament, which might straighten and stiffen the corresponding filaments. Indeed, our HS-AFM experiments with this FtsZ mutant confirmed our hypothesis and revealed a number of key differences from the wild-type (WT) protein. First, although WT filaments moved in and out of the field of view, filaments of the FtsZ L169R mutant protein appeared to be more static. This loss of mobility suggests that their kinetic polarity is perturbed. We indeed found that many filaments displayed bidirectional growth and rare, sudden shrinkage events rather than strict treadmilling behaviour (Supplementary Video 7). Second, when we analysed the shape of individual filaments at different densities, we found that mutant filaments were always straight, independent of their density. In contrast, the curvature of WT filaments decreased twofold with increasing density (Fig. 5b,c). At the same time, the contour length, that is, the length at the maximum extension of a polymer, and persistence length, which describes the rigidity of the filament^50^, were about two times higher for FtsZ L169R filaments than for FtsZ WT and further increased with their densities (Fig. 5d,e). We also found that mutant filaments showed transient filament interactions and height profile similar to the WT filaments, but with two times smaller mean filament distance (Extended Data Fig. 6a–c)^46^.

**Fig. 6:**
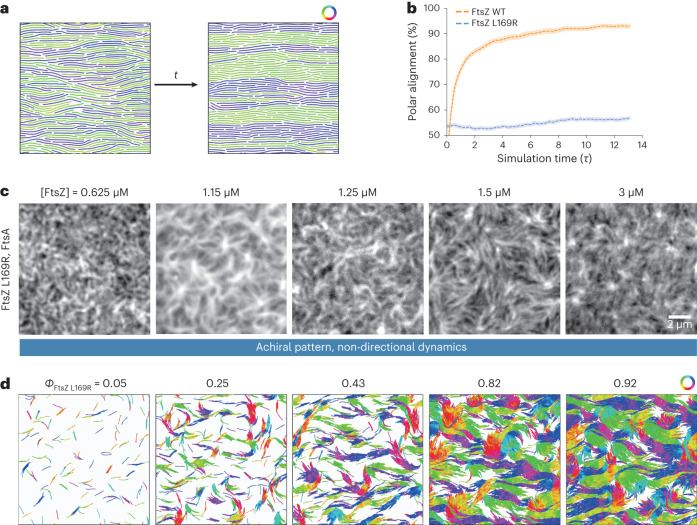
Microscopic sorting and large-scale organization of FtsZ L169R filaments. **a**, Toy simulations studying the polarity-sorting kinetics of filaments with varying intrinsic curvatures, lengths and bending rigidities—snapshot of the initial (left) and final (right) configuration of FtsZ L169R filaments at a high density of *ϕ* = 0.88. The properties of the mutant substantially slow down the polar alignment of filaments in all the studied packing fractions. Only bonds of the filaments (without the full diameter of beads) are presented for clarity. Filaments are colour coded according to the orientation of the bond vectors between beads. **b**, Fraction of the parallel (polar) alignment of filaments as a function of simulation time. The FtsZ L169R was simulated with two times higher persistence and length than WT, no intrinsic curvature and eight times lower *v*_0_ (parameters *v*_0_ = 3.625 d/τ, Pe = 300, ℱ = 20, *L*_fL169R_ = 16*d*, d*θ* = 0 rad, *ϕ* = 0.22–0.88). Although FtsZ WT prefers to align in a parallel orientation, FtsZ L169R stays aligned in a nematic fashion until the end of simulations. **c**, Representative TIRF micrographs of Alexa488-FtsZ L169R at increasing FtsZ L169R and constant FtsA concentration. We observe very static thread-like self-organization of filaments with no concentration-dependent transitions. The micrographs are representatives of at least four independent experiments (*n* = 6, 10, 6, 8 and 4 for [FtsZ] = 0.625, 1.15, 1.25, 1.5 and 3 µM). **d**, Snapshots of large-scale FtsZ L169R simulations with increasing density. The FtsZ L169R filaments were modelled with altered properties according to the HS-AFM analysis: filaments were two times longer and more rigid, had no intrinsic curvature and *v*_0_ was eight times lower, resulting in three times lower Peclet number and two times lower flexure number than FtsZ WT (parameters *v*_0_= 3.625 d/τ, Pe = 300, ℱ = 20, d*θ* = 0 rad, *L*_fL169R_ = 16*d*). FtsZ L169R exhibits a static thread-like pattern in all the densities, as in the experiment. The filaments are colour coded according to the orientation of the bond vectors between beads. Source data

Given that we previously found a predominant polar order in filament bundles in our mesoscopic simulations, we now wanted to test the effect of the filament properties of FtsZ L169R. We quantified the local filament–filament alignment in toy simulations with different filament parameters. Interestingly, we found that all the changes observed in the FtsZ L169R mutant (longer, less chiral or more persistent filaments with reduced treadmilling) went in the same direction of decreasing polar filament alignment (Extended Data Fig. 6d–g). Combining these four experimentally observed properties predicted a strong decrease in local polarity sorting (Fig. 6a,b, Extended Data Fig. 6h and Supplementary Video 8). We then decided to study the possible transitions in the organization of mutant FtsZ filaments via TIRF microscopy. FtsZ L169R polymerized into the same cytoskeletal network of filament bundles at all the tested concentrations and displayed no drastic change in architecture (Fig. 6c and Supplementary Video 9). We found that compared with the WT protein, monomer turnover and GTP hydrolysis was 2–3 times slower, which agrees with the formation of longer filaments at similar densities (Extended Data Fig. 6i–k). Importantly, we did not observe any directional flows or chirally rotating rings on the membrane surface. In addition, differential imaging did not generate directional-moving speckles, confirming that a local polar arrangement of filaments is strongly impaired (Extended Data Fig. 6l)^35^. These features were well recapitulated by the same large-scale simulations as before, but with the HS-AFM-derived properties for the mutant filaments: longer, less chiral and more persistent filaments with slower kinetics v_*0*_ (Fig. 6d, Extended Data Fig. 6m and Supplementary Video 10). Altogether, this shows that our model can provide a powerful framework to link the local microscopic structure of active filaments to the large-scale collective phases that they form as a function of density.

## Discussion

In this study, we have identified density-driven transitions in the self-organization of FtsZ filaments between three phases: a low-density disordered phase, where individual filaments treadmill in a chiral manner with few interactions; an intermediate-density phase, where curved filaments organize into polar streams and chiral vortices; and an effectively nematic phase due to density-driven filament straightening.

Using a minimal model of self-propelled filaments, we computationally analysed how their physical properties determine such transitions between the collective modes of large-scale organization. In particular, we find that the experimental observations are best recapitulated by semiflexible, curved filaments with low attraction. Indeed, although repulsive and rigid chiral filaments can self-organize into rotating rings^2^, these display not only quantitatively different frequencies and lifetimes but also qualitatively different phases with increasing density, such as integer topological defects that are not observed in the data. Highly flexible self-propelled filaments can also form stable spirals even at the single-filament level^39^, although this is not consistent with the intermediate shape fluctuations we observe here. Non-chiral filaments, such as self-propelled actin filaments on lipid membranes, can also show collective self-organized vortices, but with a different origin as they do not display chiral rotations^3^. In contrast, we show that a key feature of the collective FtsZ organization is the competition between chiral shape, active treadmilling and interfilament interactions. At intermediate densities, this results in the coexistence and interconversion of chiral vortices and travelling bands, whereas with increasing densities, the straightening of semiflexible FtsZ filaments results in a transition towards a predominant nematic-like organization.

We confirmed our theoretical predictions by visualizing the behaviour of FtsZ filaments on different spatial scales and different surface densities: the large-scale spatiotemporal dynamics of FtsZ filaments using TIRF and STED microscopy and the behaviour of individual filaments using HS-AFM. These data confirmed our theoretical prediction that the increasing density goes along with the straightening of semiflexible FtsZ filaments, which results in a transition from chiral vortices to a nematic-like organization. Furthermore, we demonstrated that a local perturbation—in this case, a specific residue in the primary sequence of a protein—can dramatically change both individual filament properties and collective self-organization.

Although much lower than that of MreB (ref. ^51^) and FtsA filaments^52^, the intrinsic curvature of FtsZ WT filaments could contribute to the correct alignment of the Z-ring perpendicular to the long cell axis particularly during the initial stages of divisome assembly^53^, when any pre-existing information at the division site is absent. Furthermore, our experimental and theoretical data suggest that weak and transient lateral interactions lacking any apparent biochemical specificity are sufficient for the alignment of treadmilling filaments on a membrane surface. Indeed, in vivo filaments in the Z-ring can move either in the same or opposite direction^54,55^, and recent experiments in *Bacillus subtilis* suggest that filament treadmilling facilitates their encounter, promoting the condensation of filaments into the Z-ring^29^. These observations are in agreement with our simulations that show that active filaments are more probable to cluster than passive filaments.

Relying on weak, non-specific interactions instead of specific residue contacts is, therefore, probably advantageous for the cell as it allows for the condensation of the Z-ring, and still permits its dynamic reorganization. The relatively low bending rigidity that we observe for FtsZ could also be the key to allow the Z-ring to adapt to the decreasing diameter of the constricting cell septum. With higher densities, filament straightening reduces the conformational flexibility and increases filament overlap, further enhancing the lateral interactions. This mechanism could, therefore, support the condensation of filaments into the tight, well-defined cytoskeletal structure that controls the remodelling of the peptidoglycan layer into two new cell poles^29,30^.

FtsZ L169R was originally described as a bundling mutant that shows enhanced lateral interactions^42,46^. Our data suggest that enhanced lateral interactions are a consequence of mutant filaments being straighter, longer and more rigid and that the closer contact between them is only a secondary effect. The lack of a preference for a polar orientation and the decreased curvature of mutant filaments agree with the observation of aberrant rings and spiral structures of FtsZ L169R found in vivo and the increased population of misshapen cells^46^. In addition, the fact that the treadmilling of this mutant is dramatically perturbed can explain why the filaments of FtsZ L169R fail to disassemble and persist at the septum after cell division^46^, whereas FtsZ WT leaves the division site before the cleavage is complete^56^.

Several experimental examples of chiral active matter have emerged in the past few years, across many different length scales. At the organismal scale, malaria parasites have been shown to have a flexible rod-like shape and actively migrate in a chiral manner with different parasites having opposite chiralities. This leads to sorting based on chirality, which is favoured by mechanical flexibility^22^. Starfish embryos, although spherical in shape, have also recently been shown to swim in a chiral manner, and collectively form crystal-like structures with an odd elastic behaviour^23^. At the cellular scale, chirality can bias the active nematic instabilities observed in confluent monolayers^57^ and biofilms^58^. This has further been proposed to arise from cytoskeletal organization, due to the polar helicoidal structure of active filaments^59^, highlighting the need to a better understanding of the collective dynamics bridging different scales. Finally, in the specific context of active filaments, although we have employed a minimal approach that aimed to capture several quantitative features of the data via a few physical ingredients, several extensions could be implemented to improve the model. For instance, it would be interesting in the future to investigate more complex models of filament treadmilling by incorporating finite lifetimes^60^ or stress-dependent polymerization. Overall, our study highlights how minimal models of active matter can bridge the scale from the local constituent property to collective self-organization, as well as provide quantitative insights into fundamental biological functions.

## Methods

### Experiments

#### Protein biochemistry

Proteins used in this study, namely, FtsZ WT, FtsZ L169R and FtsA, were purified as previously described^61^. FtsZ L169R was obtained by site-directed mutagenesis. Leucine 169 was replaced with arginine by exchanging two nucleotides (CTG→CGC). FtsZ L169R was purified as the WT protein described before.

#### Preparation of coverslips

Glass coverslips were cleaned with a piranha solution (30% H_2_O_2_ mixed with concentrated H_2_SO_4_ at a 1:3 ratio) for 60 min. The coverslips were washed with double-distilled H_2_O, 10 min sonication in ddH_2_O and again with ddH_2_O. Before supported lipid bilayer (SLB) formation, the coverslips were dried with compressed air and treated for 10 min with a Zepto plasma cleaner (Diener electronic). Then, 0.5 ml Eppendorf tubes missing the conical end were glued on the coverslips with ultraviolet glue (Norland Optical Adhesive 63) and exposed to ultraviolet light for 10 min.

#### Preparation of SUVs

1,2-Dioleoyl-*sn*-glycero-3-phosphocholine and 1,2-dioleoyl-*sn*-glycero-3-phospho-(1′-*rac*-glycerol), which were purchased from Avanti Polar Lipids, were used at a ratio of 67:33 mol%. Small unilamellar vesicles (SUVs) were then prepared as described previously^9,34,61^.

#### Preparation of SLBs for TIRF

SLBs were prepared by diluting the SUVs to 0.5 mM in a reaction buffer (50 mM Tris-HCl (pH 7.4), 150 mM KCl and 5 mM MgCl_2_) supplemented with 5 mM CaCl_2_ and incubated for 30 min at 37 °C. Finally, the SLBs were washed eight times with 200 µl reaction buffer.

#### TIRF microscopy

Experiments were performed using a Visitron iLAS2 TIRF microscope, equipped with a 63× Zeiss TIRF 1.46-numerical-aperture oil objective. The fluorophore Alexa488 was excited with a laser line at 488 nm. The emitted fluorescence from the sample was filtered using a quad-band laser filter (405/488/561/640 nm). A Cairn TwinCam camera splitter equipped with a spectral long-pass filter of 565 nm and a band-pass filter of 525/50 nm was used. Time series were recorded using a Photometrics Evolve 512 electron-multiplying charge-coupled device (512 × 512 pixels, 16 × 16 μm^2^) operating at a frequency of 5 Hz.

#### STED microscopy

STED microscopy was performed at room temperature on an inverted Expert Line STED microscope (Abberior Instruments) with pulsed excitation and STED lasers. A 640 nm laser was used for excitation and a 775 nm laser for stimulated emission. An oil-immersion objective with a numerical aperture of 1.4 (Olympus, UPLSAPO 100XO) was used for image acquisition. The fluorescence signal was collected in a confocal arrangement with a pinhole size of 0.8 Airy units. For detection, a 685/70 nm band-pass filter (Chroma, #F49-686) and a photon-counting avalanche photodiode (Laser Components, Count-T100) were used. The pulse repetition rate was 40 MHz and fluorescence detection was time gated. Data were acquired with 10 μs pixel dwell time and 30 nm pixel size for time-lapse imaging and 20 µs with 20 nm pixel size for overview images, 5.0–6.5 µW excitation laser power and 30–40 mW STED laser power. The power values refer to the power at the sample, measured with a slide power-meter head (Thorlabs, S170C). A spatial light modulator imprinted the STED phase pattern for increasing the lateral resolution. Image acquisition and microscope control were performed with Imspector software (v. 14.0.3052).

#### HS-AFM

A laboratory-built tapping mode (2 nm free amplitude, ∼2.2 MHz) HS-AFM instrument equipped with a wide-range scanner (6 µm × 6 µm) was used to visualize the dynamics of the system. BL-AC10DS-A2 (Olympus) cantilevers were used as HS-AFM scanning probes. The cantilever had a spring constant (*k*) of 0.1 N m^–1^ and a resonance frequency (*f*) of 0.6 MHz in water or 1.5 MHz in air. The dimensions of the cantilever are 9.00 µm (length), 2.00 µm (width) and 0.13 µm (thickness). To achieve a high imaging resolution, a sharpened and long carbon tip with a low apical radius was made on the existing tip of the cantilever using electron-beam deposition, as described previously^62–64^. The scanning speed varied from 0.2 to 5.0 s per frame. The number of pixels acquired were adjusted for every measurement depending on the scan size (minimum, 2 nm; maximum, about 50 nm). The in-house designed program ‘Kodec’ was used to read the data generated by HS-AFM. The software stores all the parameters, calibration and description given during the measurement and allows to load a whole folder or several videos.

#### FtsZ TIRF and STED experiments on SLBs

TIRF experiments were performed with 0.2 μM FtsA and increasing concentrations of Alexa488-FtsZ WT/L169R in 100 µl reaction buffer. Additionally, we added 4 mM ATP/GTP and a scavenging system to minimize the photobleaching effects: 30 mM d-glucose, 0.050 mg ml^−1^ glucose oxidase, 0.016 mg ml^−1^ catalase, 1 mM DTT and 1 mM trolox. FtsZ filaments were imaged by TIRF at 0.5 Hz and 50 ms exposure time.

To avoid phototoxic effects on the FtsZ filaments during STED microscopy, we accordingly adjusted our imaging setup. We replaced Alexa488 with Atto633. Furthermore, we doubled the concentrations of the scavenging solution. Finally, we changed the acquisition protocol to pixel-step-based excitation/STED cycles and introduced short breaks between the excitation cycles: 5 µs excitation/STED, 10 µs break, 5 µs excitation/STED and 10 µs break^65^. For the experiments shown, we used 0.2 µM FtsA and 1.5 µM Atto633-FtsZ. Time-lapse experiments were imaged at 0.2 Hz.

#### Preparation of SLBs for HS-AFM

SUVs were prepared as described above. Ultraflat muscovite mica layers (1.5 mm *Ø*) as the substrate were mounted on a glass stage using a standard two-component glue. The glass stage was then attached to the scanner with a thin film of nail polish. A drop of acetone was deposited on the stage–scanner interface to ensure a flat nail-polish layer. The mounted stage was dried at room temperature for about 30 min. A freshly cleaved mica layer was used as the substrate to form an SLB by depositing ~4 µl of a mix of 1 mM SUV suspension in the reaction buffer with additional 5 mM CaCl_2_. To avoid drop breakage, the scanner was flipped upside down and inserted in a custom-made mini chamber with a thin water film at the bottom (a 500 µl tube cut on the bottom and glued to a Petri dish). The drop was incubated on the stage for at least 30 min. After, the drop was exchanged 5–10 times with 5 µl of fresh reaction buffer. The stage was immediately inserted in the HS-AFM chamber containing about 80 µl of the same reaction buffer. Before the addition of proteins, HS-AFM imaging and indentation were performed to assess the quality of the SLB. When the force–distance curve showed the typical lipid bilayer indentation profile (~2–4 nm), the SLB was used in the next steps.

#### FtsZ HS-AFM experiments on SLBs

The selected proteins were added to the chamber with ATP/GTP (4 mM each) and DTT (1 mM). Optimal protein concentration and ratios were tested in a range of ~0.30–4.50 µM for FtsZ and 0.05–1.20 µM FtsA.

### Image processing and analysis

For the data analysis of TIRF and STED experiments, the videos were imported to ImageJ v.1.54^66^, and raw, unprocessed images were used. HS-AFM data were exported to ImageJ, where the post-processing, such as noise reduction and smoothing, was carried out. Noise reduction and smoothing were performed using a band-pass filter of different sizes depending on the image features. The analysis of HS-AFM videos was performed in MATLAB, using the FiberApp package^50^ (MATLAB v. 2017b). HS-AFM micrographs in the Article are raw data.

#### FtsZ intensity analysis

Saturation of FtsZ on SLBs was estimated by the titrations of FtsZ in two independent experiments. The intensity was measured after reaching equilibrium at three different positions for each concentration. Finally, we normalized the intensity values by a min–max normalization and fitted a Hill Fit $y=A+\left(B-A\right) \times (\frac{{x}^{n}}{{k}^{n}}+{x}^{n})$, where *A* is the starting point, *B* is the ending point and *n* is the Hill coefficient (Extended Data Figs. 1b and 6j).

#### FtsZ trajectory analysis

To estimate the diameter of the trajectories of FtsZ single filaments, we performed a maximum intensity projection of the selected regions of interest, showing isolated single filaments. Next, a circle was manually drawn on top of the filament trajectory and its measured diameter (Fig. 1b).

#### Differential imaging to measure directional autocorrelation and velocity

To quantify the directional flows of FtsZ filaments, we used a previously developed automated image analysis protocol^35^. Growing ends of FtsZ filaments were tracked to compute their directional autocorrelation. The autocorrelation curves were best fitted assuming a fast and slow decay, whose rates were extracted by fitting a two-phase exponential decay *y* = *a*1 × e^(–*b*1×*t*)^ + *a*2 × e^(–*b*2×*t*)^, where *a*1 and *a*2 are the starting points and *b*1 and *b*2 are the fast and slow decay rates (Fig. 1c and Extended Data Fig. 1d).

#### FRAP analysis

To obtain the recovery half-time of the bleached areas, we used a Jython macro script for ImageJ (Image Processing School 8 Pilsen 2009) to fit the fluorescence recovery with *I*(*t*) = *a*(1 – e^–*bt*^), where *I*(*t*) is the intensity value corrected for photobleached effects. FRAP experiments were acquired with an exposure time of 50 ms and an acquisition time of 1 Hz (Extended Data Figs. 1e and Fig. 6i).

#### Collision angle analysis from STED videos

The angle of FtsZ filament collision was measured using the angle tool in ImageJ. Three points, corresponding to the incoming FtsZ and the centre point of the collision, were manually set. The subsequent alignment (parallel or antiparallel) was manually evaluated (Extended Data Fig. 2b,c).

#### Quantification of GTP hydrolysis rate of FtsZ

We used the EnzChek kit to measure the GTPase rate of FtsZ (Thermo Fisher, E6646). The protein was buffer exchanged into a phosphate-free reaction buffer and diluted to 5 µM in 20× reaction buffer supplemented with 2-amino-6-mercapto-7-methylpurine riboside (MESG) and purine nucleoside phosphorylase (PNP). The reaction was incubated for 15 min to remove traces of free phosphate. Then, 200 µM GTP was added and the production of free phosphate was measured (Extended Data Fig. 6k).

### Ring analysis

#### Ring density

The number of rings in a given field of view was manually counted after averaging every 40 s of a time-lapse video (Fig. 2d,e and Extended Data Fig. 4a).

#### Ring diameter, thickness and lifetimes

A walking average was performed over five frames of a time-lapse video, and an intensity profile was drawn across the ring diameter.

We subtracted the background and fitted a double Gaussian function with the Python v.3.9.13 SciPy module^67^. The ring width is defined as the full-width at half-maximum averaged over the two peaks. The outer diameter of rings was reported and compared with simulations (Fig. 2f and Extended Data Fig. 4f,h). The ring lifetimes were obtained by preparing a kymograph along the diameter of a ring and then measuring the length of two parallel vertical lines.

### Single-filament analysis

HS-AFM videos were imported to FiberApp^50^ and the filaments were traced. We used the A* pathfinding algorithm with 100 iterations, open-contour type and the following parameters: alpha = 10, beta = 10, gamma = 20, kappa1 = 20, kappa2 = 10. We extracted the persistence length (*L*_p_) from the mean-squared end-to-end distance ${y}^{2}=4 \times {L}_{{\rm{p}}}\times \left[x-2\times {L}_{{\rm{p}}}\times \left(1-{{\rm{e}}}^{-\frac{x}{2\times {L}_{{\rm{p}}}}}\right)\right]$. The contour length was extracted from the average length of all the frames within a specific FtsZ density. The local curvature was directly extracted from the *x*–*y* coordinates of the filaments. Subsequently, we averaged the local curvatures of each filament to obtain one curvature value per filament at different densities (Figs. 4b and 5c–e). To obtain reliable values for curvature, persistence and contour lengths, we analysed all the filaments within ten subsequent frames at the corresponding packing fractions. The mean result of all the filaments is shown as dots (Figs. 4b and 5c–e).

### Numerical simulations

The simulations of FtsZ filaments are based on the self-propelled worm-like chain model^39^, extended for polymer chirality (Fig. 2a). A filament is represented by *N* + 1 beads with radius *r*_0_ connected by *N* stiff bonds and chiral bending potentials. To minimize friction between the polymers, the bond length is chosen to be equal to *r*_0_, leading to overlaps of neighbouring beads and filament length *L*_f_ = *Nr*_0_. The overdamped equation of motion is, therefore, given by

$$\gamma \frac{{\rm{d}}{\bf{r}}_{i}}{{{\rm{d}}t}}={\bf{\nabla }}_{i}V+{\bf{F}}_{{\rm{p}}}^{i}+{\bf{F}}_{{k}_{{\rm{B}}}T}^{i},$$

where *r*_*i*_ are the coordinates of the beads, *γ* is the friction coefficient, ${F}_{{k}_{{\rm{B}}}T}^{i}$ is the thermal noise force (modelled as white noise with zero mean and variance 4*k*_B_*Tγ*/δ*t*, where δ*t* is the simulation timestep and *k*_B_*T* is the effective temperature) and *V* is the potential energy, which comprises both intra- and interfilament interactions:

$$V={V}_{{{\rm{bond}}}}+{V}_{{{\rm{bend}}}}+{V}_{{{\rm{pair}}}},$$

where the first term is a harmonic bond potential that penalizes filament stretching, that is,

$${V}_{{\rm{bond}}}(r)=\frac{1}{2}{k}_{{\rm{bond}}}\mathop{\sum }\limits_{i=1}^{N-1}{\Big(\,{\mathbf{r}}_{i,i+1}-{r}_{0}\Big)}^{2},$$

whereas the second term is a harmonic bending potential that penalizes filament bending, that is,

$${V}_{{{\rm{bend}}}}\left(\theta \right)=\frac{1}{2}\frac{{k}_{{{\rm{bend}}}}}{d}\mathop{\sum }\limits_{i=1}^{N-2}{\left({\theta }_{i}-{\theta }_{0}\right)}^{2},$$

where ${\vec{r}}_{i,i+1}$ is the bond vector between the neighbouring beads, *θ*_*i*_ is the angle between the neighbouring bonds and *θ*_0_ represents the equilibrium rest angle (*k*_bond_ and *k*_bend_ are the corresponding spring constants, the latter being defined in units of energy × length for consistency with units of bending rigidity for one-dimensional beams; note that we have rescaled the effective bead diameter by $d=2\sqrt{2}{r}_{0}$, which we have used below to non-dimensionalize all the lengths). The rest angle is related to the intrinsic filament bending angle d*θ* = π − *θ*_0_, where non-zero d*θ* implies chirally curved filaments, whereas the *k*_bond_ parameter is large enough to keep the bond length in the polymer constant (see Extended Data Table 1 for list of parameter values). Finally, filaments interact via a soft Lennard–Jones potential, which accounts for excluded volume interactions and potentially a middle-range attraction as

$$\begin{array}{rcl}{V}_{{\rm{pair}}} & = & \mathop{\sum }\limits_{i}\mathop{\sum }\limits_{j > i}{v}_{{\rm{pair}}}({r}_{i,\,j})\\ {v}_{{\rm{pair}}}({r}_{i,\,j}) & = & \left\{\begin{array}{cc}4\epsilon \Big[{\Big(\frac{\sigma }{{r}_{i,\,j}}\Big)}^{4}-{\Big(\frac{\sigma }{{r}_{i,\,j}}\Big)}^{2}\Big]+{\Delta {{v}}}_{{\rm{pair}}}({r}_{i,\,j}), & \quad{r}_{i,\,j} < {r}_{{\rm{cut}}}\\ \qquad\qquad\qquad\qquad\qquad\qquad\qquad 0, & \quad{r}_{i,\,j} < {r}_{{\rm{cut}}}\end{array}\right.\end{array},$$

where *r*_*i*,*j*_ is the distance between the positions of beads *i* and *j* (which can belong to any filament, excluding beads *j* which are nearest and next-nearest neighbours on the same filament as bead *i*), *ϵ* is the depth of the potential well, *σ* = 2*r*_0_ is the characteristic distance of interaction and *r*_cut_ is the cutoff distance of the potential (see Extended Data Table 1 for list of parameter values). The potential is shifted by a linear pair potential Δ*v*_pair_, to ensure that the potential and its derivative smoothly goes to zero at the cutoff $\frac{\partial {v}_{\rm{pair}}}{\partial r}\left({r}_{\rm{cut}}\right)=0$. When accounting only for repulsive interactions, we use *r*_cut_ consistent with the interaction minimum $\sqrt{2}\sigma$ (see Extended Data Table 1). Here ${F}_{{\rm{p}}}^{i}$ is the active self-propulsion force that mimics the filament treadmilling (acting tangentially along the bonds of the polymer with force per unit length *f*_p_, or alternatively for more intuitive units, resulting in a single-filament speed of *v*_0_):

$${\bf{F}}_{{\rm{p}}}^{i}={f}_{{\rm{p}}}{{\bf{r}}}_{i-1,i}=\gamma {v}_{0}\frac{{{\bf{r}}}_{i-1,i}}{\left|{{\bf{r}}}_{i-1,i}\right|}$$

for 1 < *i* ≤*N*. Note that to ensure chiral self-propulsion, the first bead *i* = 1 has a modified chiral force of ${\bf{F}}_{{\rm{p}}}^{i}=\gamma {v}_{0}\frac{{{\bf{r}}}_{i,i+1}+{{\bf{c}}}}{\left|\,\,{{\bf{r}}}_{i,i+1}+{{\bf{c}}}\right|}$, where ${\bf{c}}=(\cos {\rm{d}}\theta ,\sin {\rm{d}}\theta )$. The simulations are performed in two dimensions and employ periodic boundary conditions.

### Details of simulation setup and parameter exploration

HOOMD-blue v. 2.9 (ref. ^68^) was used to run the simulations, with in-house modifications of the chiral worm-like chain model. Specifically, to ensure the chirality of the polymers, an asymmetric bending potential was used, having a signed curvature (always calculated in the direction from head to tail of the filament).

The simulation parameters and results are reported in a dimensionless form, where the length is measured in units of the effective bead diameter *d* (defined as the interaction minimum $\sqrt{2}\sigma$), energies in units of the thermal energy *k*_B_*T* and time in units of the rotation period of a single isolated FtsZ WT filament without noise *τ* (for convenience when comparing to experiments). The equations of motion were numerically integrated using the Euler scheme with timestep δ*t* = 1.8 × 10^–5^*τ*. The dynamics of filaments was mainly governed by two dimensionless numbers (note that *f*_p_ is defined as a force by unit length), namely, the Peclet number

$${{\rm{Pe}}}=\frac{{f}_{{\rm{p}}}{L}_{{\rm{f}}}^{2}}{{k}_{{\rm{B}}}T}$$

and the flexure number

$${\mathcal{F}}=\frac{{f}_{{\rm{p}}}{L}_{{\rm{f}}}^{3}}{{k}_{{{\rm{bend}}}}}.$$

Unless stated otherwise, the simulation parameters to model FtsZ WT are summarized in Extended Data Table 1.

To compare with experiment, the reduced simulation units were recalculated with constants 1*d* = 50 nm (corresponding to filament length FtsZ WT *L*_f_ = 400 nm and filament curvature of 3.48 rad µm^–1^) and *τ* = 78.5 s (considering a ring with a diameter of 1 μm and treadmilling speed of 0.04 μm s^–1^). Although the dimensions of individual FtsZ molecules are around 10 nm, our coarse-grained model assumes membrane-recruited filaments with an effective width of 50 nm, motivated by experimental observations^42^ (Extended Data Fig. 6a). We define the packing fraction as *ϕ* = *NN*_f_d*r*_0_/*L*^2^, where *N*_f_ refers to the number of filaments and *L* is the box size.

To check if our explored parameter space is reasonable, we performed a few sanity checks. We computed the persistence length of filaments in our simulations from the distribution of local filament curvatures in intermediate density^56^. Here *L*_p_ was obtained by fitting a Gaussian function to the local curvature data and extracting its variance: ${L}_{{\rm{p}}}=\frac{1}{{\sigma }^{2}\Delta s}$, where *σ* is the standard deviation of the Gaussian function and Δ*s* is the filament contour spacing^69,70^. The resulting values of *L*_p_ = 6,753 nm for FtsZ WT (Extended Data Fig. 4k) and *L*_p_ = 11,527 nm for FtsZ L169R (Extended Data Fig. 6m) agree well with our HS-AFM analysis (Fig. 5d). We also checked the order of magnitude of the Peclet numbers of FtsZ WT in our simulations (Extended Data Fig. 4l) by comparing the variability of single-filament trajectories (short simulations ran for 0.5*τ*) to experimental trajectories from the TIRF data (Fig. 1b). Next, we ran simulations corresponding to the collision histogram from STED (Extended Data Fig. 2b,c) to check how is the alignment of colliding filaments affected by the intrinsic curvature of filaments, and we found a good agreement with the experimental data for chiral filaments with a curvature of 2.1–3.5 rad µm^–1^ (Extended Data Fig. 4n,o). Finally, we wanted to check how the velocity of single filaments changes with increasing density. In agreement with differential imaging and single-particle tracking (Extended Data Fig. 1f,g), the velocity of single filaments decays only slightly. Altogether, these model data comparisons support that we can strongly constrain the region of the parameter space based on the available dataset.

The simulations of active filaments were initialized in a nematic configuration (each filament was placed on the lattice in a straight configuration randomly oriented either left or right). The initial time needed for the equilibration of the system (4*τ*) is discarded in the analysis. Parameter screening and subsequent simulations, which were focused on the molecular details (temporal interconversion of rings and bands (Fig. 2c), comparison of filament packing in active and passive systems and topological defects (Fig. 3b–g) and polarity sorting (Fig. 6a,b)) were performed with a box size of *L* = 42*d*, whereas all the reported large-scale patterns were simulated in a box of size *L* = 212*d*.

To get a well-mixed state of non-chiral filaments with lower *F*_p_ (Fig. 6a,d), the system was initiated in low density (*ϕ* = 0.25 and lower) on a lattice in a nematic configuration with high thermal noise force (Pe = 100). The denser systems were initialized with enlarged boxes of density *ϕ* = 0.25, and after mixing of the filaments, these systems were downscaled and equilibrated in a stepwise manner, to reach a high-density system with effectively random initial conditions.

The large-scale simulations (Figs. 2b and 6d) were run for 20*τ*, whereas the small systems (where the temporal interconversion of individual rings and bands was quantified (Fig. 2c)) were run for 40*τ*. Finally, polarity sorting simulations (Fig. 6a,b) were run for 10*τ* and analysed from the initial timestep. Each combination of parameters was run in at least ten repeats for small system sizes and five repeats for larger systems.

### Simulation analysis

Unless stated otherwise, the simulation frames were analysed with a frequency of 0.2*τ* after the equilibration. The average filament curvature (Figs. 3a,b and 4b and Extended Data Fig. 3b,c) was computed by first calculating the mean curvature of each filament and then averaging it over a single snapshot of the system. The resulting distribution of the average curvatures consists of pooled data over both multiple simulation time points and simulation repeats.

To automatically detect rings in the system, the instantaneous centres of rotation were computed for each filament in each analysed snapshot. These points were subsequently clustered using the freud library^38,57^ with the distance threshold related to the density of the system (cutoff defined as half of the average distance between filaments: $\frac{L}{2\sqrt{{N}_{{\rm{f}}}}}$). Clusters with more than ten members and a normalized radius (given by the radius of gyration/cluster size) lower than 0.1*d* were considered to be ring centres. To filter out unclosed rings, the normalized polarity (given by the norm of the vector summing the filament orientations/cluster size) of filaments belonging to a ring was computed and had to be lower than 0.25*d*. Finally, to improve ring detection in high density, we filtered vortices without a ring-like shape, that is, having the lower density of filaments in the centre of the vortex—local filament density in the ring centre had to be lower than the packing fraction of 0.5 (cutoff radius 2*d*) (Extended Data Fig. 4i). Since stable rings did not show significant overall translation, we performed the additional clustering of ring centres in time with a distance cutoff of 3*d* to track the rings in time and get a lifetime of the rings (estimated by the subtraction of last and first snapshots of each observed ring; Extended Data Fig. 4b–e). This clustering was also used to remove falsely detected ‘rings’ by removing all the clusters of size lower than three members (Extended Data Fig. 4j). All the cutoffs of this analysis were chosen and validated by visual comparison to the simulation videos in all the studied densities.

To calculate the average density of rings in simulations, the number of rings in a snapshot was divided by the total area (Fig. 2d,e and Supplementary Fig. 4a). The diameter of the rings was estimated by calculating the average distance of filaments from the centre of the ring (Fig. 2f and Supplementary Fig. 4f,g).

The polarity alignment (Fig. 6b and Extended Data Fig. 6d–h) was analysed by computing all the neighbouring beads (not belonging to the same filament, with the same distance threshold *r*_cut_ used in the pair potential) in the system using the freud library and classifying the neighbour-relative orientation as either parallel or antiparallel based on their angle (<90­°, parallel; >90°, antiparallel).

The local density of active and passive filaments (Fig. 3b and Extended Data Fig. 5a) was analysed by first collecting the position of the central bead of each filament and then calculating the number of monomer neighbours around this position with a cutoff of 2*d* (although other cutoffs gave similar trends). The number of neighbours was then recalculated into the packing fraction and presented as a binned scatter plot.

The density fluctuations (Fig. 3c and Extended Data Fig. 5a) were analysed by iteratively splitting the simulation box into smaller boxes with increasing sizes and calculating the number density of monomers in each box. For each box size, the mean and standard deviation of the number density were plotted to determine the scaling of density fluctuations.

The topological defects in high-density simulations (Fig. 3d–g) were analysed by first collecting the positions and orientations of each bond in a simulation box and then calculating the nematic director field using all the positions (grid size 4.7*d* corresponding to 225 nm). The edges of the simulation box were extended by an additional grid point to track the defects also at the periodic boundaries. From the nematic director field, we extracted the winding numbers of every even grid point by integrating anticlockwise around overlapping 3 × 3 grid point loops, assigning charges −1/2, +1/2, +1 and −1 for integral values of –π, π, 2π and −2π, respectively (Extended Data Fig. 5f). Every simulation frame was checked if its total charge was equal to zero (which was the overwhelming majority, with rare errors emerging due the occasional gaps even at the highest filament density); only these frames were used for the analysis of defect density. Python package Trackpy^71^ was used to track the topological defects to analyse their lifetime and mean-squared displacement (Extended Data Fig. 5c–e). We separately tracked each type of defect before recombining the results. The maximum displacement between frames was set as three grid points and we merged trajectories if only one frame was missing between a given tracked defect. Tracks shorter than three frames were flagged as random defects and excluded from the subsequent analysis, if the total topological charge in the simulation frame was equal to zero after this step.

### Protein structure prediction

Protein modelling was performed with AlphaFold-Multimer v2 (refs. ^48,72^) implemented in Google Colab using the filament structure of *S. aureus* FtsZ PDB 3VOB as a template.

### Reporting summary

Further information on research design is available in the Nature Portfolio Reporting Summary linked to this article.

## Online content

Any methods, additional references, Nature Portfolio reporting summaries, source data, extended data, supplementary information, acknowledgements, peer review information; details of author contributions and competing interests; and statements of data and code availability are available at 10.1038/s41567-023-02218-w.

### Supplementary information

Supplementary Information Captions for Supplementary Videos 1–10.

Reporting Summary

Supplementary Video 1 TIRF time-lapse videos of FtsZ WT.

Supplementary Video 2 STED time-lapse videos of FtsZ WT.

Supplementary Video 3 Visual phase diagram of large-scale FtsZ patterns.

Supplementary Video 4 Temporal coexistence of rings and bands.

Supplementary Video 5 Topological defects at high densities.

Supplementary Video 6 HS-AFM time-lapse videos of FtsZ WT.

Supplementary Video 7 HS-AFM time-lapse videos of FtsZ L169R.

Supplementary Video 8 Polar sorting of filaments with different properties.

Supplementary Video 9 TIRF time-lapse videos of FtsZ L169R.

Supplementary Video 10 Simulations of FtsZ L169R.

### Source data

Source Data Fig. 1 Statistical source data.

Source Data Fig. 2 Statistical source data.

Source Data Fig. 3 Statistical source data.

Source Data Fig. 4 Statistical source data.

Source Data Fig. 5 Statistical source data.

Source Data Fig. 6 Statistical source data.

Source Data Extended Data Fig. 1 Statistical source data.

Source Data Extended Data Fig. 2 Statistical source data.

Source Data Extended Data Fig. 3 Statistical source data.

Source Data Extended Data Fig. 4 Statistical source data.

Source Data Extended Data Fig. 5 Statistical source data.

Source Data Extended Data Fig. 6 Statistical source data.

## Data Availability

Source data are provided with this paper. Raw microscopy images and full datasets are available via the online repository of the Institute of Science and Technology Austria at 10.15479/AT:ISTA:13116.

## Extended data

**Extended Data Table 1:** Summary of the used simulation parameters

Parameter	Notation	Chosen value for FtsZwt (simulation units)	Parameter screening		Fitting/Constraint
			range	incre-ment	
Self-propulsion speed	v_0_	29 *d*/τ	fixed to correspond to treadmilling speed 40 nm/s		
Bonding potential	k_bond_	1000 *k*_*B*_*T*/*d*^2^	fixed parameter (to keep the bond length constant)		
Timestep	*δt*	1.8×10-5 τ	fixed parameter (small enough for numerical stability)		
Friction coefficient	*γ*	1	fixed parameter		
Attraction cutoff	*r*_*cut*_	1.7 *d* (1 *d* for no attraction)	fixed parameter		
Filament length	*L*_*f*_	8 *d*	8–40	8	chosen to correspond to filament length 400 nm and thickness 50 nm
Peclet number (changed by thermal noise)	*Pe*	900	10–10000	200	chosen according to variation of single filament trajectories (Fig. 1b, Extended Data Fig. 4l, m)
Bending rest angle	*d*θ	0.06 *rad*	0.0–0.07	0.01	chosen according to single filament trajectories (Fig. 1b) and collision histogram (Extended Data Fig. 4n)
Packing fraction	*Φ*	0.05–0.95	0.05–0.95	~ 0.05	Values chosen according to the titration curve (Extended Data Fig. 1b)
Flexure number (changed by bending rigidity)	${\mathcal{F}}$	40	5–200		free parameter
Interaction strength	*ε*	0.1 *k*_*B*_*T* (same for no attraction)	0.1–1.5	0.15	free parameter

## Code availability

All code used to produce these results is available from the corresponding authors upon reasonable request.
