# Supplementary material for: Chiral and nematic phases of flexible active filaments
Source: Nat Phys. 2023 Oct 9;19(12):1916–26. doi: 10.1038/s41567-023-02218-w (PMC10709145; doi:10.1038/s41567-023-02218-w)
Supplement: Supplementary file 1 — Captions for Supplementary Videos 1–10. [file 41567_2023_2218_MOESM1_ESM.pdf]

nature physics

Article

https://doi.org/10.1038/s41567-023-02218-w

Chiral and nematic phases of flexible active filaments

In the format provided by the authors and unedited

Supplementary Video legends

Supplementary Video 1: TIRF time-lapse movies of FtsZ WT

TIRF time-lapse movies of Alexa488-FtsZ WT at different concentrations ([FtsA] = 0.2µM). With increasing FtsZ concentration, the pattern changes from rotating rings and directional moving filament bundles to a more nematic pattern. Movies were acquired at 0.5 frame per second and correspond to **Fig. 1a**.

Supplementary Video 2: STED time-lapse movies of FtsZ WT

STED time-lapse movies of Atto633-FtsZ WT. The FtsZ concentration was 1.25µM and FtsA 0.2µM. The experiments were recorded at 1 frame every 4-6s, where the acquisition rate depends on the field of view. The movies display the co-existence of rings and bundles of FtsZ filaments. The movies correspond to **Extended Data Fig. 2a**.

Supplementary Video 3: Visual phase diagram of large-scale FtsZ patterns

Large scale FtsZ patterns (L = 212 d) with varying filament flexibility (measured by flexure number $\mathcal{F}$, vertical axis) and packing fractions (horizontal axis). Filaments are colored according to the orientation of the bond vectors between beads. We observe ring-like self-organization of rigid filaments ($\mathcal{F}$ = 5), spatial coexistence of chiral rings and polar bands in regime of semiflexible filaments ($\mathcal{F}$ = 40) and disordered patterns with flexible filaments ($\mathcal{F}$ = 200). Movies correspond to **Fig. 2b**.

Supplementary Video 4: Temporal coexistence of rings and bands

Temporal coexistence of chiral rings and polar bands in a small system (L = 42 d) of intermediate density (ϕ = 0.5) and filament flexibility ($\mathcal{F}$ = 40). With increasing density, the ring state becomes unstable and filaments organize only in bands. Filaments are colored according to the orientation of the bond vectors between beads. Movie corresponds to **Fig. 2c** and **Extended Data Fig. 3c**.

Supplementary Video 5: Topological defects at high densities

Topological defects in high filament density (ϕ =0.95) and small system size (L = 42 d). The rigid filaments ($\mathcal{F}$ = 5) form stable integer (+1 and -1) topological defects,

semiflexible filaments ($\mathcal{F}$ = 40) form only nematic defects (+½ and -½) due to the filament straightening and flexible filaments ($\mathcal{F}$ = 200) enter a "chaotic phase" with increased number of defects due to their broad curvature distribution. The simulation movie corresponds to 120 s in real time. Only bonds of the filaments are presented for clarity and colored according to the orientation of the bond vectors.

Supplementary Video 6: HS-AFM time-lapse movies of FtsZ WT

HS-AFM movies of FtsZ WT filaments. As the density of FtsZ WT filaments on the supported lipid bilayer increases, they become less dynamic and straighter. At high densities, the filaments show a nematic order with topological defects. Time-lapse movies were acquired with 3 and 2 frames per second. The movies correspond to **Fig. 4a,c.**

Supplementary Video 7: HS-AFM time-lapse movies of FtsZ L169R

HS-AFM videos of FtsZ L169R filaments. As the density of FtsZ L169R increases, but the filaments remain straight and static. At high densities, the filaments pack extremely tight together. Images were acquired with 3 and 2 frames per second. The movies correspond to **Fig. 5b.**

Supplementary Video 8: Polar sorting of filaments with different properties

Polar sorting of filaments with properties of FtsZ WT and FtsZ L169R in two different densities. FtsZ L169R is 2x longer and more rigid, non-chiral and has lower self-propulsion than FtsZ WT, resulting in 3x lower Peclet number and 2x lower flexure number. Only bonds of the filaments are presented and colored according to the orientation of the bond vectors.

Supplementary Video 9: TIRF time-lapse movies of FtsZ L169R

TIRF time-lapse movies of Alexa488-FtsZ L169R at increasing concentrations ([FtsA] = 0.2μM). FtsZ L169R does not form rings as seen for FtsZ WT and the pattern appears less dynamic. Movies were acquired at 0.5 frame per second and correspond to **Fig. 6c**.

Supplementary Video 10: Simulations of FtsZ L169R

Simulations of increasing concentration of FtsZ L169R. The mutant filaments are non-chiral, 2x longer and more rigid than FtsZ WT and are self-propelled with 8x lower speed,

resulting in Pe = 200 and $\mathcal{F}$ = 20 and filament persistence length 2x longer than FtsZ WT. FtsZ L169R does not form rings and self-organizes into less dynamic pattern. Movies correspond to **Fig. 6d.**
